# Association of Red Cell Distribution Width and Mean Platelet Volume With Disease Activity in Rheumatoid Arthritis Patients

**DOI:** 10.7759/cureus.56908

**Published:** 2024-03-25

**Authors:** Haseeb Ahmed Khan, Sana Haseeb Khan, Zaid Tayyab, Saba Saif, Saima N Khan, Sana Musaddiq

**Affiliations:** 1 Rheumatology, Dorset County Hospital, Dorchester, GBR; 2 Hematology, Al Aleem Medical College, Lahore, PAK; 3 Rheumatology, Fatima Memorial Hospital College of Medicine and Dentistry, Lahore, PAK; 4 Medicine, CMH Lahore Medical College and Institute of Dentistry, Lahore, PAK; 5 Internal Medicine, Allama Iqbal Medical College/Jinnah Hospital, Lahore, PAK; 6 Internal Medicine, Jinnah Hospital, Lahore, PAK

**Keywords:** erythrocyte sedimentation rate, disease activity, mean platelet volume, rheumatoid arthritis, red cell distribution, association

## Abstract

Background: The inner layer of the synovial joints is the primary target of rheumatoid arthritis, or RA, a chronic systemic inflammatory disorder that is linked to increasing disability, early mortality, and economic hardships. The objective is to determine the association of red cell distribution width (RDW) and mean platelet volume (MPV) with disease activity in RA.

Material and methods: A retrospective study was conducted between July 2021 and January 2022 in the outpatient rheumatology clinics at Gulab Devi Teaching Hospital. In this study, 100 consecutive participants with a diagnosis of RA fulfilling the ACR/EULAR 2010 classification criteria were enrolled. Patient’s records were reviewed for age, gender, length of illness, smoking status, treatment history, current treatment regimen, concomitant medications, rheumatoid factor (RF), anti-cyclic citrulline peptide (anti-CCP) antibodies, and extra-articular manifestations. Laboratory investigations were reviewed for complete blood count including RDW and MPV, ESR, CRP, liver, and renal functions. Disease activity score DAS 28-ESR was used to quantify disease activity. To determine the relationship between different parameters and the RDW and MPV, linear regression research was conducted.

Results: According to the DAS28 score, 12% of patients were in remission, 9% had low, 34% had moderate, and 45% had high disease activity. DAS28 score was 5.01±1.72 (2.45-9.32) and RDW was 16.18±4.42. There was a strong positive correlation (r = 0.653) of RDW with the DAS28 score and it was statistically significant (p<0.001). MPV was 11.30±2.09 fL. There was a moderately positive correlation (r = 0.366) of MPV with the DAS28 score and it was statistically significant (p<0.001).

Conclusion: Conclusively, both RDW and MPV are positively related to disease activity in patients with RA. These can be used as a simple tool for assessing disease activity and guiding the treatment.

## Introduction

The inner layer of the synovial joints is the primary target of rheumatoid arthritis (RA), a chronic inflammatory disease that is linked to increasing disability, early mortality, and economic hardships [1]. Among 0.5% and 1% of the adult population suffer from RA. Three times as many women as males are afflicted by the illness [2]. The key components of diagnosis and routine follow-up include clinical symptoms, examination, laboratory tests, and imaging. Autoantibodies and inflammatory indicators of rheumatoid factor (RF), C-reactive protein (CRP), erythrocyte sedimentation rate (ESR), and others are tested in a laboratory setting [3]. Hemogram parameters have lately been suggested in a number of international research as indicators of inflammation. In addition to their significance for hemostasis, scholars have suggested that they represent the burden of inflammation. Red cell distribution width (RDW) and mean platelet volume (MPV) are two of these hemogram measures [4]. RDW is a parameter that shows the fluctuation in RBC size and volume and is incorporated in the hemogram with automated analyzers. According to a number of investigations, a person's RDW may be a good indicator of their overall health condition and, more precisely, of how inflammatory they may be. Inflammation can affect many parameters that could lead to an increase in size heterogeneity, including membrane deformability and the half-life of RBC circulation [5]. Red blood cell distribution width rises in response to any procedure that causes reticulocytes to be released into the bloodstream [6]. RDW has also been connected to both single as well as multiple organ failure, indicating the level of anisocytosis in several illnesses, including liver and kidney failure, diabetes mellitus, cancer, heart disease, and venous thromboembolism. Furthermore, RDW is considered a separate risk factor for death. We do not, however, know if a higher RDW in RA represents a risk factor or should just be viewed as an epiphenomenon of an inherent biological and metabolic instability including aberrant red blood cell survival as well as defective erythropoiesis [7-11]. It was thus discovered in some research about the activity of the illness or its repercussions, and it may thus be elevated in autoimmune disorders. Comparable to erythrocyte sedimentation rate or C-reactive protein, it is now plausible to propose RDW as a possible biomarker [12].

The rate of production of platelets in the marrow and the size of circulating platelets are observed in MPV, which can also serve as an indication of inflammation intensity and the activation of platelets. MPV has been studied in several inflammatory disorders as a probable pointer of disease activity [13]. Tumors, atherosclerosis, and RA have all been linked to the survival of platelet receptors, including GPIb/IX/V, CD40, and selectins [14]. Immune complexes interact with Fc receptors in disorders like systemic lupus erythematosus, or SLE, triggering platelets. In the inflamed synovium of RA, platelets are a well-known generator of prostaglandins. Platelet-derived vesicles containing IL-1 are widely distributed in synovial fluid and induce the formation of inflammatory mediators by synovial fibroblast. Furthermore, in the inflamed synovium, vascular permeability is increased due to serotonin produced by platelets [15].

It is still unknown if MPV levels and RDW are interrelated to clinical disease activity indicators of RA. The literature is replete with information about the correlation between inflammation and both RDW and MPV levels. The therapeutic use of these criteria is still uncertain, despite their affordability and ease of use, and the findings of disparate investigations about the levels of MPV in a range of inflammatory illnesses are inconsistent [4,16]. Therefore, the goal of this research was to determine the MPV levels and RDW in patients with RA and to investigate if these are linked to the disease activity.

## Materials and methods

A retrospective study was conducted between July 2021 and January 2022 in the outpatient rheumatology clinics at Gulab Devi Teaching Hospital. In this study, 100 consecutive participants with a diagnosis of RA fulfilling the ACR/EULAR 2010 classification criteria were enrolled. All the included patients were above 18 years of age. Patients younger than 18 years, having other autoimmune conditions, liver and renal disease, malignant diseases, hematological disorders (iron deficiency anemia or having had blood transfusions within the previous four months), cerebrovascular disease, metabolic and cardiovascular diseases, hyperthyroidism or hypothyroidism, pregnancy, or six months during the postpartum period were excluded.

The patient’s records were reviewed for age, gender, length of illness, smoking status, treatment history, current treatment regimen, concomitant medications, RF, anti-cyclic citrulline peptide (anti-CCP) antibodies, and extra-articular manifestations. Laboratory investigations were reviewed for complete blood count including RDW and MPV, ESR, CRP, liver, and renal functions. Disease activity score DAS 28-ESR was used to quantify disease activity [17,18]. Disease activity was categorized as follows: remission (<2.6), low disease activity (LDA) (2.6-3.2), moderate disease activity (MDA) (>3.2-5.1), and high disease activity (HDA) (>5.1).

The standard deviation and mean were used to show the age, BMI, and length of illness, which were all normally distributed. Percentages and numbers were used to represent categorical variables. The means of continuous variables were linked using the independent t-test (sometimes known as the student's t-test). The Chi-square test was used to associate the categorical variables. By dividing the independent variable into two binary levels and comparing it to a single dependent variable (in this scenario, the RDW), binary logistic regression analysis was used to get the OR (odd ratio). To assess the relationship between different parameters and the MPV and RDW, a linear regression analysis was conducted. The statistical analysis was conducted using SPSS version 22 (IBM Corp., Armonk, NY, USA), with a significance threshold of p-value < 0.05.

## Results

A total of 100 patients were studied. The mean age of the patients was 37.09±10.53 years and the mean BMI was 26.13±3.63 kg/m^2^. The study population included 37% males and 63% females. Active smoking was present in 32% of the patients while 12% patients were ex-smokers. Disease duration ranged from 01 to 22 years with a mean of 8.72±5.04 years (Table 1).

**Table 1:** Disease-related data of the patients. DAS - Disease Activity Score, RDW - Red Cell Distribution Width, MPV - Mean Platelet Volume, fL - fililiter.

Variable	Value (n=100)
Disease severity, n (%)	
Remission	12 (12.0%)
Low disease activity	9 (9.0%)
Moderate disease activity	34 (34.0%)
High disease activity	45 (45.0%)
DAS28 ESR	5.01±1.72 (2.45 – 7.32)
RDW (%)	16.18±4.43 (8 – 25)
Correlation	r = 0.653, p<0.001
MPV, fL	11.30±2.09 (8 – 17)
Correlation	r = 0.366, p<0.001

According to the DAS28 score, 12% patients were in remission, 9% had low, 34% had moderate and 45% had HDA. DAS28 score was 5.01±1.72 (2.45 - 7.32) and RDW was 16.18±4.42. There was a strong positive correlation (r = 0.653) of RDW with the DAS28 score and it was statistically significant (p<0.001). MPV was 11.30±2.09 fL. There was a moderately positive correlation (r = 0.366) of MPV with the DAS28 score and it was statistically significant (p<0.001) (Table 2).

**Table 2:** Demographic details of the patients. BMI - Body mass index, n - number of patients.

Variable	Value (n=100)
Age, years	37.09 ± 10.53 (19 - 64)
BMI, kg/m^2^	26.13 ± 3.63 (21 - 36)
Gender, n (%)	
Male	37 (37.0%)
Female	63 (63.0%)
Residence, n (%)	
Urban	54 (54.0%)
Rural	46 (46.0%)
Smoking, n (%)	
Yes	32 (32.0%)
No	56 (56.0%)
Ex-smoker	12 (12.0%)
Disease duration, years	8.72 ± 5.04 (01 - 22)

## Discussion

This study focused on finding the correlation of RDW and MPV with the disease activity of RA by using the DAS-28 scoring system. The findings of our study showed a positive correlation and thus like many previous studies provided vital proof that MPV and RDW can be used to assess the disease severity of RA.

In literature, there have been multiple studies in recent times that have similar results to our study of note is the case-control study done by Al-Rawi et al. who concluded that there was a significant difference in the value of RDW in cases as compared to controls thus showing positive correlation [19]. Enhanced RDW during RA can be clarified by the fact that RA is an autoimmune prolonged condition that frequently coexists with anemia, which can raise RDW [20]. Another explanation is that inflammation can potentially have an impact on RDW [21]. Other studies have demonstrated an increase in RDW in a variety of inflammatory diseases [22,23].

According to Tecer et al. [21] RDW was comparable to C-reactive protein and ESR indicating inflammatory activity, and it was considerably greater in RA. Moreover, RDW and DAS28 were also correlated. RDW was found to be correlated with inflammatory factors and autoantibody levels in RA by Yunchun et al. [24]. They proposed that this connection might be related to elevated oxidative stress and an inherent pro-inflammatory state, both of which are associated with poor erythrocyte development. RDW has also been shown to have a significant role in the proinflammatory and proatherogenic condition of RA and may be a valuable indicator for better characterizing the progression of the disease in RA patients. Nevertheless, Rodríguez-Carrio et al.'s investigation could not find a link between DAS-28 and RDW [25]. This could be connected to the study's smaller sample size as well as statistical power. In a study done recently, He et al. looked into RDW as a probable lab variable for tracking inflammation in RA. They found that patients with RA had higher levels of RDW than control group patients and that these levels were associated with both cytokines (anti-inflammatory and inflammatory). Based on these findings, they proposed that RDW level could be a useful inflammatory indicator for observing inflammation and the progression of the disease in RA patients [6].

In terms of the subsequent issue, a great deal of research has been done on the function of platelets in RA and inflammation, and the findings have demonstrated the critical role that platelets play in inflammatory response [26-28]. Platelet markers, also known as indices, are easily derived from CBC and represent platelet function. The most significant markers are mean platelet volume and PDW. The present study's results, along with those of other earlier investigations, supported the hypothesis that MPV and RDW would therefore represent the degree of inflammation. Meanwhile, these findings ran counter to those of other studies [29].

## Conclusions

Conclusively, both RDW and MPV are positively associated with disease activity in patients with RA. These are readily available, cost-effective tools that can be used to predict disease activity and guide management. Further studies in larger cohorts are needed for the validation of these tools.
